# Influence of the Polymerization Parameters on the Porosity and Thermal Stability of Polymeric Monoliths

**DOI:** 10.3390/ma17122860

**Published:** 2024-06-12

**Authors:** Małgorzata Maciejewska

**Affiliations:** Department of Polymer Chemistry, Institute of Chemical Sciences, Faculty of Chemistry, Maria Curie-Skłodowska University, Gliniana 33, 20-614 Lublin, Poland; mmacieje@umcs.pl

**Keywords:** polymeric monoliths, porous structure, thermal properties, thermogravimetry

## Abstract

Rigid porous polymeric monoliths are robust, highly efficient, versatile stationary phases. They offer simple preparation and convenient modification provided by a whole range of synthesis factors, e.g., starting monomers, cross-linkers, initiators, porogens, polymerization techniques, and temperature. The main aim of this study was to synthesize polymeric monoliths and determine the correlation between polymerization parameters and the porosity and thermal stability of the obtained materials. Polymeric monoliths were synthesized directly in HPLC columns using *N*-vinyl-2-pyrrolidone (NVP) and 4-vinylpiridine (4VP) as functional monomers, with trimethylolpropane trimethacrylate (TRIM) serving as the cross-linking monomer. During copolymerization a mixture of cyclohexanol/decane-1-ol was used as the pore-forming diluent. Polymerization was carried out at two different temperatures: 55 and 75 °C. As a result, monoliths with highly developed internal structure were synthesized. The value of their specific surface area was in the range of 92 m^2^/g to 598 m^2^/g, depending on the monomer composition and polymerization temperature. Thermal properties of the obtained materials were investigated by means of thermogravimetry (TG). Significant differences in thermal behavior were noticed between monoliths synthesized at 55 and 75 °C. Additionally, the poly(NVP-*co*-TRIM) monolith was successfully applied in GC analyses.

## 1. Introduction

Porous polymeric monoliths are an intriguing category of materials that can be an excellent alternative to classical beads. The concept of applying a single piece of porous material as a separation medium was first suggested in the 1950s [1]. However, a new type of stationary phase was successfully applied after 50 further years of extensive research [2,3,4,5,6,7,8]. Depending on the type of substrate, monoliths can be divided into three categories: organic polymer monoliths, inorganic monoliths (mainly silica monoliths), and hybrid monoliths [9,10,11]. Inorganic silica monoliths are predominantly based on alkoxysilane. Polymeric monoliths are mostly prepared by a radical polymerization. The polymerization mixture, containing functional monomer, cross-linker, porogens, and an initiator is placed into the column or capillary. The used porogens can be solvating or non-solvating diluents for the polymer. Also, the soluble polymers, or the mixture of soluble polymers and solvents, can be applied as pore-forming agents. The polymerization reaction is triggered by elevated temperature or UV light. At a certain degree of conversion, the process of phase separation occurs [12,13,14,15]. The classic mechanism of pore formation involves the initially homogeneous polymerization mixture splitting into solid and liquid phases. The solid phase is composed of a cross-linked polymer network, whereas the solvent and unreacted comonomers make up the liquid phase [16,17]. The phase separation is induced by the increase in cross-linking degree (*ν*-syneresis) or by the change in the polymer–solvent interaction (macro- or microsynersis). In microsyneresis, the liquid phase is dispersed in the polymer gel phase, whereas in macrosyneresi, nuclei are formed. The nuclei possess a higher than average cross-linking density and are surrounded by a liquid phase. They can react with each other through free double bonds, forming macrogels. The free space between macrogels generates mesopores, while micropores are present between nuclei. The occurrence of macrogel agglomerates leads to macropores. This fundamental mechanism of pore formation remains appropriate regardless of the polymerization technique. However, in the case of monolith synthesis, some differences occur compared with beads preparation. The nuclei and their agglomerates have a higher density than the polymerization medium and, consequently, can sediment at the bottom of the mold. They form a loose, very porous arrangement. At this stage the nuclei maintain their individuality. Nevertheless, during polymerization they come into contact and the structure becomes more cohesive. The constant formation of new polymer chains leads to the strong interconnection of the nuclei. The polymerization process continues both within the monomer solution present above the growing rod, and within the mixture that permeates the huge pores of the growing rod. The formation of a large number of new nuclei occurs. Subsequently, the very large pores disappear in the latter stages of polymerization and the nuclei lose their individuality. As a consequence, a decrease in both specific surface area and pore volume takes place. 

The porous properties of synthesized monoliths can be adjusted by the proper choice of the pore-forming solvent [18,19,20,21,22,23,24]. For the formation of a macroporous structure, phase separation is necessary as an a priori condition. During polymerization the cross-linked nuclei separate from the solution as a result of their limited solubility in the polymerization medium. The phase separation depends strongly on the thermodynamic quality of the porogens. In the synthesis of porous monoliths two-component solvent systems are commonly used. A higher content of thermodynamically poor solvent leads to product with larger pores, due to the earlier phase separation. The new phase mainly swells with the monomers as they are better solvents for the growing polymer than the porogen. Therefore, the monomer concentration in the swollen nuclei is much higher than in the local solution. As a consequence, the polymerization process predominantly occurs within the enlarged nuclei, rather than within the solution. The formed globules are quite large and, accordingly, the free voids between them are also large. While the thermodynamic quality of the solvent increases, a good solvent can compete with monomers in the solvation process of nuclei. The monomer concentration in the nuclei is considerably lower and, consequently, the globules grow slower. For that reason, monoliths synthesized in thermodynamically good solvents have small pores. Hence, the porosity of the monoliths can be controlled by the solvation of the polymer chain in the polymerization medium. In recent decades, an excessively broad selection of porogens was applied in the synthesis of porous monoliths. Typically they have been a binary solvent mixture, e.g., dodecanol and cyclohexanol; 1,4 butanediol and propanol; or tetrahydrofuran and decanol. Based on the widely approved recipe, most monoliths are polymerized with a monomer to solvent ratio of 40:60 wt%. 

What is extremely important is that nearly any monomer may be used in monolith synthesis. The mold utilized in polymerization contains only one phase. Consequently, hydrophilic and reactive monomers that are inappropriate for suspension polymerization can be also applied for monolith formation. One can find monoliths based on acryladmide, 2-acryloamido-2-methyl-1-propanesulfonic acid, glycidyl methacrylate, chloromethylstyrene, 1-vinyl-2-pyrrolidone, or 2-vinyl-4,4-dimethylazlactone, The monomers incorporate a wide range of functionalities into the polymeric network. Unfortunately, the selection of cross-linking monomers is quite limited. The family of applied cross-linkers mainly includes divinylbenzene, ethylene glikol dimethacrylate, *N*,*N*′ methylenebisacryloamide, and trimethylolpropane trimethacrylate [25,26,27,28,29,30,31,32,33,34]. The cross-linking monomer concentration in the polymerization mixture can vary to a great extent. Increasing the proportion of cross-linker to functional monomer leads to a decrease in pore sizes. This phenomenon is connected with early phase separation and early formation of highly cross-linked nuclei. A high degree of cross-linking of the nuclei diminishes the adsorption of monomers and reduces their tendency to coalesce. The number of individual nuclei remains high and, consequently, the free voids between them are small. The resulting pore size distribution is shifted toward smaller pores. 

The majority of porous monoliths are designed to work at ambient temperatures. This may lead to the impression that their thermal stability is less important. Nevertheless, certain applications like gas chromatography (GC) or catalysis (e.g., automobile exhaust abatement reactors) need elevated temperatures. The GC analysis becomes shorter at higher temperatures and the rate of catalysis reaction is higher. Thus, the thermal stability of polymeric monoliths is an important feature. 

The aim of this study was to synthesize thermally stable porous polymeric monoliths and to determine the influence of polymerization temperature on the textural and thermal properties of the obtained products. To achieve this goal, *N*-vinyl-2-pyrrolidone (NVP) and 4-vinylpiridine (4VP) were used as functional monomers, while trimethylolpropane trimethacrylate (TRIM) served as the cross-linking monomer. The binary solvent mixture (cyclohexanol/decan-1-ol) was applied as pore-forming agent. The polymerization was conducted directly in the standard columns for high performance liquid chromatography (HPLC) at 55 °C and 75 °C. The monolith properties were correlated with the polymerization temperature. 

## 2. Materials and Methods

### 2.1. Chemicals

*N*-vinyl-2-pyrrolidone, α,α’-azoisobutyronitrile (AIBN), and decan-1-ol were from Fluka (Buchs, Switzerland), whereas trimethylolpropane trimethacrylate and 4-vinylpyridine were purchased from Sigma Aldrich (Steinheim, Germany). Cyclohexanol was obtained from Acros (Geel, Belgium). Methanol was from Merck (Darmstad, Germany). Ethanol, butan-1-ol, pentan-1-ol, and heksan-1-ol were supplied by POGh (Gliwice, Poland).

### 2.2. Monolith Synthesis

The synthesis were performed within the HPLC columns (100 mm × 4.6 mm), provided with a heating jacket. All of the polymerization mixtures containing functional monomer, cross-linker, porogens, and initiator were purged with nitrogen for 15 min before polymerization. Then, they were transferred into the stainless steel column. The columns were sealed with PTFE plugs and put into the polymerization vessel in which the polymerization occurred. Thermostated water circulating through the jacket provided the required temperature (75 °C for M1, M2, and M3 monoliths and 55 °C for M1B, M2B, and M3B monoliths). The polymerization proceeded for 24 h. After completion of the polymerization, the plugs were replaced by column-end fittings and the rods were thoroughly cleaned with methanol using a dual piston, high performance constant pressure pump. The experimental conditions are summarized in Table 1.

### 2.3. Measurement Methods

A Bruker Tensor 27 FTIR spectrometer (Ettlingen, Germany). was used to obtain attenuated total reflectance infrared spectra in the spectral range of 4000–600 cm^−1^ with a resolution of 4 cm^−1^ (32 scans per spectrum).

The elemental analysis of the synthesized copolymers was performed with the use of a Perkin-Elmer 2400 CHN analyzer (USA).

For the detailed characterization of porous structure of the monoliths, the nitrogen adsorption–desorption measurements were made using a volumetric adsorption analyzer ASAP 2405 (Micrometrics Inc., Norcross, GA, USA).The measurements were conducted at −196 °C. The specific surface area was calculated using the Brunauer–Emmet–Teller method (*S_BET_*). The total pore volume (*V*) and pore diameters (D*_BJH_*) were obtained from the desorption branch of the isotherm using the Barrett–Joyner–Halenda procedure.

The internal structure of the monoliths was imaged using a scanning electron microscope (SEM) Duall BeamTM, Quanta3D FEG (Fei Company, Hillsboro, OR, USA).

Thermal properties of the investigated monoliths were assessed on the basis of thermogravimetry and differential scanning calorimetry. TG measurements were performed with a Netzsch STA 449 F1 Jupiter thermal analyzer (Selb, Germany) in the range of 30–750 °C in inert (helium) and oxidative (synthetic air) atmospheres. The characteristic temperatures of *T*_5%_, *T*_20%_, and *T*_50%_ mass losses, as well as the final decomposition temperature (*FDT*) were gauged on the basis of the TG curves, whereas the differential TG (DTG) curves were used to establish the temperature of the maximum rate of mass loss (*T*_max_) for individual decomposition stages. 

Chromatographic measurements were carried out on a Dani GC 1000 gas chromatograph (Dani, Milan, Italy) equipped with a packed injector (220 °C) and a thermal conductivity detector (TCD, 220 °C). Helium, at a flow-rate of 50 mL/min, was used as carrier gas. The samples were manually injected using a 1 μL syringe (SGE, North Melbourne, Australia). Measurement were conducted at 140 °C and 200 °C. 

## 3. Results

In the synthesis of porous polymeric monoliths, two functional monomers containing nitrogen in their structures were used. In order to incorporate pyrrolidone units into a polymeric matrix, *N*-vinyl-2-pyrrolidone was applied. NVP contains a highly polar amide group which has hydrophilic and polar-attracting properties. Additionally, it possesses apolar methylene groups in both the backbone and the ring, thereby conferring hydrophobic properties. Polymers derived from NVP possess numerous advantageous properties, including, but not limited to, hydrophilicity, no toxicity, biocompatibility, and complexability. However, NVP has considerable solubility in water. Consequently, synthesis of NVP-based copolymers using suspension, emulsion, or multi-step swelling polymerization is heavily restricted [35,36,37]. In the synthesis of polymeric monoliths, this problem can be easily circumvented as the polymerization proceeds in one phase mold [38,39]. In the case of 4-vinylpyrridine, the presence of a vinyl group and a heteroaromatic ring is responsible for its high reactivity. It can be easily used in heterogeneous system as well as in monolith production [40,41,42]. 

**Figure 1:**
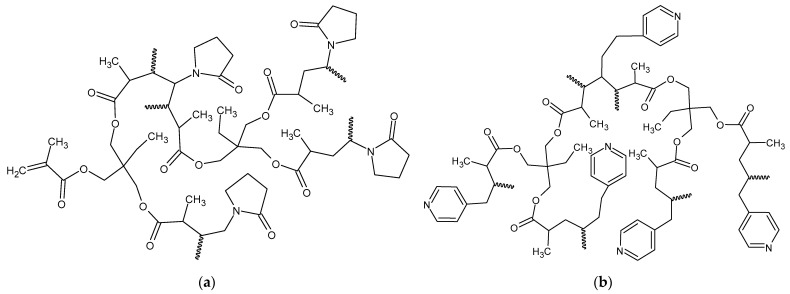
The schematic chemical structure of (**a**) poly(NVP-*co*-TRIM) and (**b**) poly(4VP-*co*-TRIM) monoliths.

The schematic chemical structures of the obtained copolymers are presented in Figure 1. For comparative purposes poly(TRIM) monolith was also prepared.

In the current research, the molar ratio of functional monomer to cross-linker in the polymerization mixture was 1:1. The actual amount of functional monomer in the polymeric matrix was calculated on the basis of elemental analysis. This procedure was possible due to the presence of nitrogen in the monomers. As can be seen from Table 2, nearly all of the 4VP was incorporated into copolymer. In the case of NVP, the value was slightly lower. What is interesting, is that in the case of monoliths synthesized at higher temperatures (M1, M2), the determined nitrogen content and, consequently, the amount of functional monomer was increased. In all cases the cross-linking was slightly above 50%.

**Figure 2:**
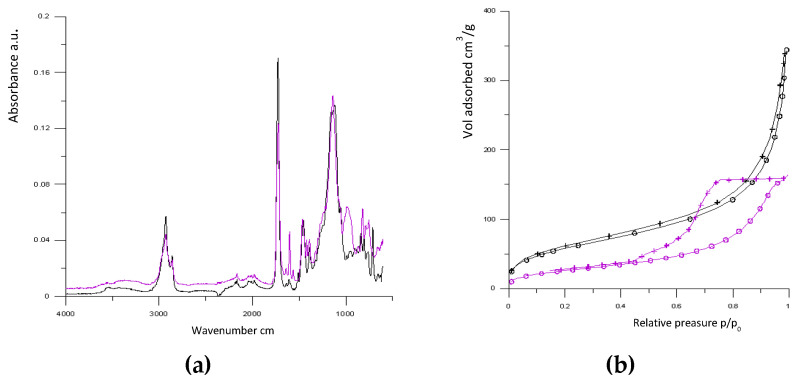
ATR-FTIR spectra (**a**) of poly(4VP-*co*-TRIM) obtained at 55 °C (purple line) and 75 °C (black line) and the adsorption (◦)/desorption (×) isotherms (**b**) of nitrogen determined for poly(4VP-*co*-TRIM) synthesized at 55 °C (purple line) and 75 °C (black line).

Polymerization was carried out at two different temperatures: 55 and 70 °C. The polymerization temperature had a large effect on the properties of the obtained porous materials. Figure 2a shows ATR-FTIR spectra of poly(4VP-*co*-TRIM) obtained at 55 and 75 °C. Apart from bands connected with C=C and C=N stretching vibrations of the pyridine ring (bands between 1599 cm^−1^ and 1558 cm^−1^), the carbonyl group (C=O stretching vibration at 1745 cm^−1^ and COC stretching vibration at 1173 cm^−1^), and alkyl and alkylene groups, (2962–2853 cm^−1^), in the case of the monolith obtained at 55 °C, bands associated with unreacted double bounds (C=C stretching vibration at 1660 cm^−1^ and =C-H deformation vibration at 1005) are clearly visible. This means that at lower temperatures the monomer conversion was not fully completed. 

The polymerization temperature also had a large impact on pore formation. Free-radical polymerization consists of several consecutive steps. Generally, the initiation has the highest activation energy. For this reason, the initiation reaction is the most temperature dependent. The initiator decomposition, the number of growing radicals, and the total polymerization rate are greater at higher temperatures. Thus, at elevated temperatures, a higher number of nuclei is formed at once, all of which swell with the residual monomers. The reservoir of monomers is quickly consumed as the nucleation rate is much faster than the swelling rate. The sizes of growing nuclei remain quite small. Consequently, the interstitial voids between nuclei are also small. If the polymerization temperature is lower, the reaction rate is significantly lower. The number of nuclei is also smaller and they have more time to swell in the remaining monomers, and their growth is maintain by polymerization that is continued within them. As a consequence, the free voids that are free between nuclei are larger, and the distribution of pore sizes shifts towards larger pores. Hence, the dimensions of pores exhibited an inverse correlation with the polymerization temperature. Figure 2b presents the adsorption/desorption isotherms of nitrogen determined for poly(4VP-*co*-TRIM) synthesized at 55 and 75 °C. According to the IUPAC, they can be classified as type IV, which characterizes mesoporous adsorbents. The isotherms of the monolith obtained at 55 °C showed hysteresis loops that could be attributed to capillary condensation. Hysteresis loops may exhibit different shapes. As regards the discussed sample, the hysteresis loop was broad and could be classified as H2 type. Type H2 loops can be found in many porous adsorbents, and this kind of loop occurs when there is a difference in mechanisms between condensation and evaporation. This phenomenon takes place in pores with narrow necks and wide bodies (ink-bottle shape) or when the porous material has an interconnected pore network. Along with the increasing the polymerization temperature the adsorption and desorption branches of the isotherm start to merge. Calculated on the basis of nitrogen adsorption/desorption isotherms, basic parameters of porous structures are presented in Table 3. As can be seen, the polymerization temperature is an especially effective means of controlling the internal structure. It enables the preparation of porous monoliths with different specific surface areas, pore volumes, and pore sizes from a single polymerization mixture. Monoliths obtained at 75 °C (M1, M2, and M3) were characterized by much more developed specific surface area comparing with their counterparts synthesized at 55 °C (M1B, M2B, and M3B). A similar observation was made by Svec and Frechet [43]. They examined poly(glycidyl methacrylate–co-ethylene dimetacrylate) monoliths synthesized at various temperatures using a cyclohexanol/dodecanol mixture or toluene as porogenic solvents. It was found that in higher temperatures, more primary nuclei were formed. Their dimensions were small and, consequently, their specific surface area was large. On the other hand, the changes in total pore volume were insignificant. The polymerization temperature in monolith synthesis was also discussed in later works of Svec’s group [2,3,4,5,6,7].

**Figure 3:**
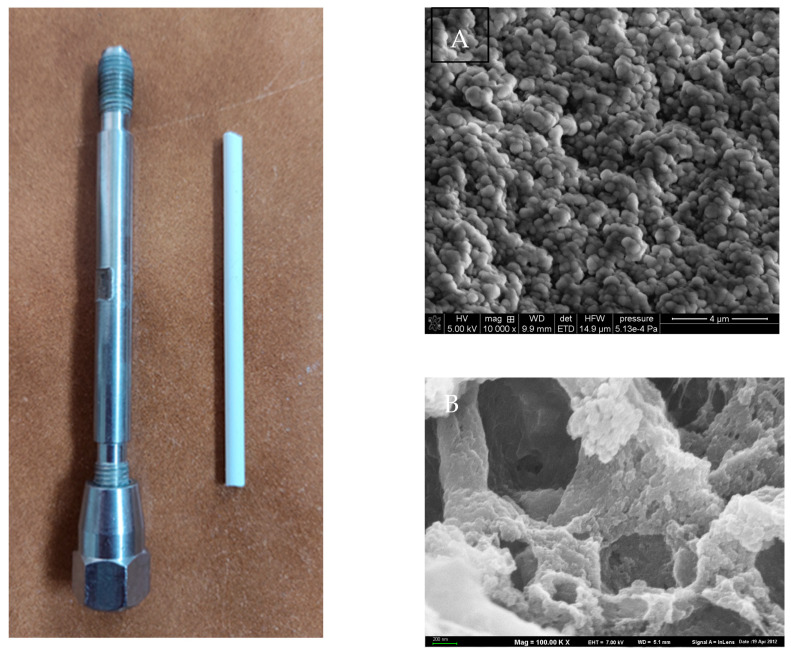
Images of the HPLC column along with M1 monolith (**left**) and internal structure of the monolith (**right**). Zoom (**A**) 10,000× and (**B**) 100,000×.

At elevated temperatures, the polymerization reaction proceeded more rapidly, and a greater number of growing chains were converted into individual globules instead of being entrapped by the primary nuclei. These globules were small, which meant that their surface was quite large. Consequently, the specific surface area of the monoliths synthesized at 75 °C was over two times larger than that of those synthesized at 55 °C. In contrast to the specific surface area, the changes in total pore volume were not as significant. What is important is that, at higher temperatures, the pore-size distribution profile was shifted toward smaller diameters. This phenomenon is also visible in the SEM images (Figure 3).

**Figure 4:**
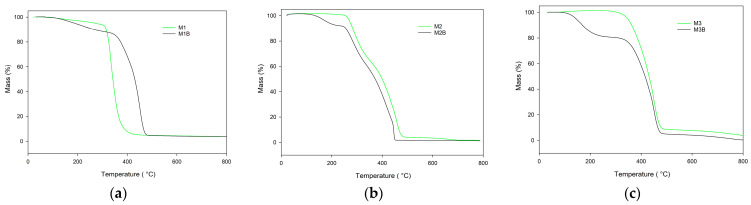
TG curves of the investigated monoliths obtained in helium atmosphere: (**a**) poly(NVP-*co*-TRIM), (**b**) poly(4VP-*co*-TRIM), and (**c**) poly(TRIM).

The most-developed porous structure was observed for the monolith composed of pure cross-linker (M3) at 75 °C. Its specific surface achieved almost 600 m^2^/g. In a system where a high percentage of cross-linker phase separation occurs early, however, the adsorption of remaining monomers and swelling of newly formed nuclei is limited by their high crosslinking degree. Additionally, high cross-linking density reduces the tendency to coalescence. The average pore size decreases and consequently these materials are not permeable to liquids at acceptable pressure. Nevertheless, they could be used to support the immobilization or the catalysts. The majority of the catalytic processes are carried out at elevated temperatures. Consequently, the applied material must demonstrate considerable thermal resistance. In this research, the thermal behavior of the investigated monoliths was assessed on the basis of thermogravimetry. Figure 4 displays TG curves of the monoliths obtained in the helium atmosphere. On their basis the characteristic temperatures were evaluated and they are presented in Table 4. As can be seen, the best thermal resistance was found in the M3 monoliths composed of the pure cross-linker (TRIM). The initial decomposition temperature of this monolith significantly exceeded 300 °C. Incorporation into the polymeric matrix functional monomer considerably decreased the thermal resistance, regardless its type (NVP or 4VP).

**Figure 5:**
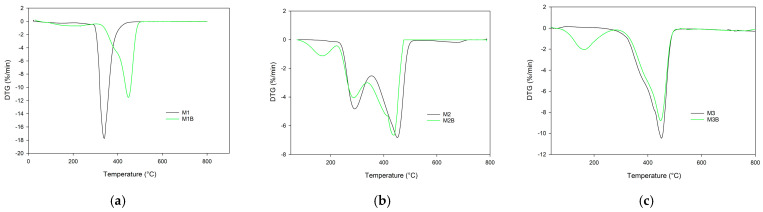
DTG curves of the investigated monoliths obtained in helium atmosphere: (**a**) poly(NVP-*co*-TRIM), (**b**) poly(4VP-*co*-TRIM), and (**c**) poly(TRIM).

The initial decomposition temperatures of the 4VP-*co*-TRIM (M1) and NVP-*co*-TRIM (M2) copolymers were practically the same (slightly above 270 °C). The main difference in their thermal behaviors can be seen in the DTG curves (Figure 5). The thermal decomposition of 4VP-*co*-TRIM (M1) monolith proceeded in one step with the maximum at 338 °C. In the case of the NVP-*co*-TRIM (M2) monolith, two distinct peaks, with maxima at 290 °C and 455 °C, are clearly visible. This phenomenon indicates that in the structure of M2 monoliths, homopolymeric domains of poly(NVP) and poly(TRIM) can be found. What is more, the *T*_max2_ of NVP-*co*-TRIM copolymer corresponded with the temperature of maximum decomposition of the poly(TRIM) monolith.

Despite the alternations in thermal behavior of chemically different monoliths, the most profound distinctions were visible between materials obtained at different polymerization temperatures. Reduction in the synthesis temperature from 75 °C to 55 °C resulted in earlier decomposition onset. The most profound difference was visible in the case of poly(TRIM) monoliths (M3 and M3B). The initial decomposition temperature declined from 342 °C (M3) to only 142 °C (M3B). Also, the decomposition profile had changed its course. For the monolith obtained at 55 °C, the decomposition process proceeded in two distinct steps with the maxima *T*_max1_ at 232 °C and *T*_max2_ at 506 °C. Additional decomposition steps were also observed for the remaining materials synthesized at 55 °C. This phenomenon can be associated with the lower degree of conversion of these monoliths.

The data collected in the synthetic air atmosphere indicated that thermal resistance of the monoliths was much worse in an oxidative atmosphere. The initial decomposition temperatures (IDTs) of all of the monoliths under study were lower when compared with their counterparts determined in an inert atmosphere (Table 5).

**Figure 6:**
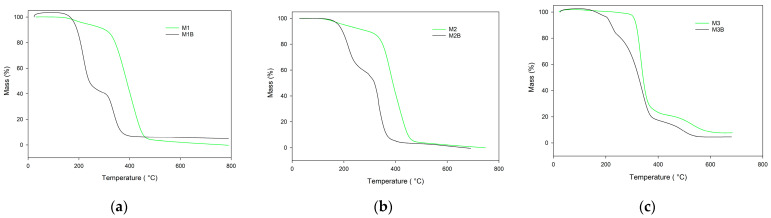
TG curves of the investigated monoliths obtained in synthetic air atmosphere: (**a**) poly(NVP-*co*-TRIM), (**b**) poly(4VP-*co*-TRIM), and (**c**) poly(TRIM).

What is interesting, is that a difference was also visible between M2 and M3 monoliths while in helium their IDTs were the same. In an oxidative atmosphere, the M1 monolith containing an aromatic ring showed better resistance than the M2 monolith based on pyrrolidone units. What is worth noticing, is that when synthesized from pure cross-linker, the M3 monolith exhibited the highest thermal stability among the obtained materials. Similar to the helium atmosphere, monoliths synthesized at 55 °C exhibited a lower thermal stability in synthetic air compared with those obtained at 75 °C. This phenomenon was especially visible in the case of poly(TRIM) material (Figure 6).

**Figure 7:**
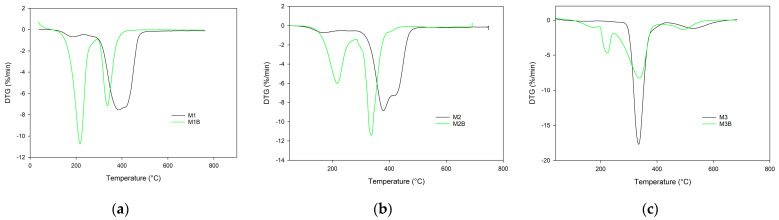
TG curves of the investigated monoliths obtained in synthetic air atmosphere: (**a**) poly(NVP-*co*-TRIM), (**b**) poly(4VP-*co*-TRIM), and (**c**) poly(TRIM).

The IDT of M3B monolith was over 100 °C lower than that of M3. Also, the remaining indicators of its thermal stability were reduced. What is more, the thermal degradation of M3B monolith obtained at 55 °C proceeded in two steps with maxima at 227 and 339 °C, as distinct from M3, with one maximum at 335 °C (Figure 7).

**Figure 8:**
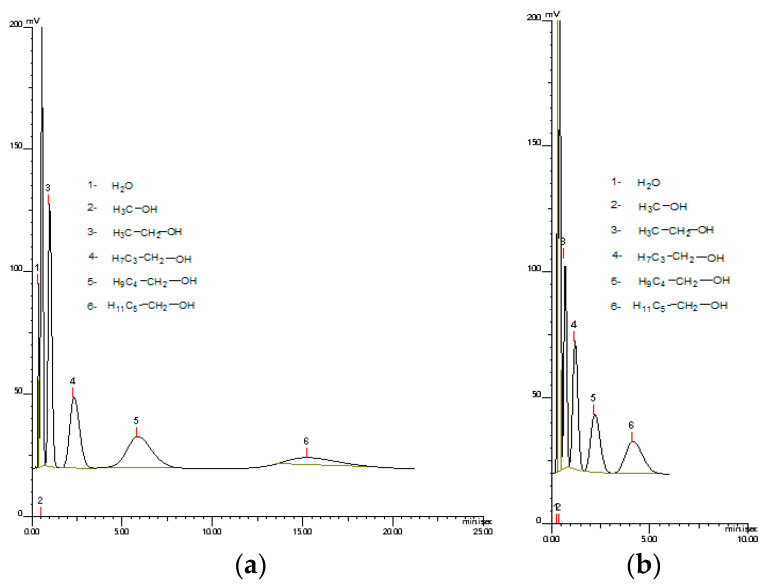
GC chromatograms of alcohols separation at 140 °C (**a**) and 200 °C (**b**) conducted on poly(NVP-*co*-TRIM) copolymer.

The good thermal resistance of the synthesized monoliths opens up the possibility of their utilization in gas chromatography. Additionally, the presence of pyrrolidone units in M2 copolymers provides hydrophilic properties. It enables the interaction of poly(NVP-*co*-TRIM) copolymer with polar compounds, e.g., alcohols. Figure 8 presents the chromatograph of mixture of aliphatic alcohols conducted at 140 °C and 200 °C. As can be seen, at higher temperatures the time required for alcohol separation was much shorter and the peaks were not diffused. Therefore, monoliths that are thermally stable are more advantageous for GC techniques.

## 4. Conclusions

The main aim of this research was to synthesize and characterize porous, thermally stable polymeric monoliths. To achieve this goal, NVP and 4VP were applied as functional monomers, while TRIM served as cross-linker. Polymerization was carried out directly in an HPLC column with the use of a cyclohexanol/decane-1-ol mixture as the binary pore-forming agent. As a result, monoliths with highly porous internal structure were obtained. Their structural and thermal properties were strongly correlated with the polymerization temperature. Materials synthesized at 75 °C possessed more developed specific surface areas and higher thermal resistance compared with their counterparts obtained at 55 °C. At the same time, their pore diameters were shifted towards smaller values. The porous monoliths could be used in various adsorption techniques and their good thermal stability is highly advantageous. Moreover, the polymerization temperature can be efficient tool for synthesis of materials with divergent properties from one established polymerization mixture, as it requires no changes in the reaction-mixture composition.

## Figures and Tables

**Table 1:** The designations and experimental parameters of the monoliths’ synthesis.

Monolith	Monomers/mmol			Diluents/mL		Temperature /°C
	TRIM	NVP	4VP	Cyclohexanol	Decan-1-ol	
**M1**	10	-	10	10	3.5	75
**M1B**	10	-	10	10	3.5	55
**M2**	10	10	-	10	3.5	75
**M2B**	10	10	-	10	3.5	55
**M3**	15	-	-	15	5	75
**M3B**	15	-	-	15	5	55

**Table 2:** Nitrogen and cross-linker content in the synthesized monoliths.

Monolith	Nitrogen Content in the Copolymer (%)		Cross-Linker Content (%)
	Calculated	Determined	
**M1**	3.17	3.14	50.5
**M1B**	3.17	3.08	51.4
**M2**	3.13	2.98	52.4
**M2B**	3.13	2.74	56.3
**M3**	-	-	100
**M3B**	-	-	100

**Table 3:** The main parameters of the porous structure of the monoliths.

Monolith	Specific Surface Area *S_BET_* (m^2^/g)	Pore Volume *V* (cm^3^/g)	Pore Diameter *D_BJH_* (nm)
**M1**	212	0.483	24
**M1B**	92	0.248	45
**M2**	556	0.830	20
**M2B**	227	0.530	44
**M3**	598	0.871	24
**M3B**	203	0.392	2/19/32

**Table 4:** TG and DTG data of the monoliths obtained in helium atmosphere.

Monolith	*T*_5%_ (°C)	*T*_20%_ (°C)	*T*_50%_ (°C)	*T*_max1_ (°C)	*T*_max2_ (°C)	*T*_max3_ (°C)	FDT
**M1**	273	325	342	338	-	-	484
**M1B**	166	341	396	221	447	-	467
**M2**	274	304	399	290	455	-	483
**M2B**	183	283	372	164	283	434	457
**M3**	342	385	431	450	-	-	512
**M3B**	142	311	415	232	506	-	497

**Table 5:** TG and DTG data of the monoliths obtained in synthetic air atmosphere.

Monolith	*T*_5%_ (°C)	*T*_20%_ (°C)	*T*_50%_ (°C)	*T*_max1_ (°C)	*T*_max2_ (°C)	*T*_max3_ (°C)	FDT
**M1**	225	343	388	183	387	-	460
**M1B**	183	206	240	217	337	-	401
**M2**	199	350	389	166	378	406	461
**M2B**	180	214	318	216	337	-	394
**M3**	311	326	341	335	-	-	650
**M3B**	208	257	326	227	339	-	548

## Data Availability

The data presented in this study are available on request from the corresponding author.
